# Quantitative analysis of e-health's impact on health systems

**DOI:** 10.3389/fdgth.2025.1674015

**Published:** 2025-10-31

**Authors:** Heba Alqurashi

**Affiliations:** Public Health Department, College of Health Sciences, Saudi Electronic University, Dammam, Saudi Arabia

**Keywords:** e-health, healthcare systems, technology adoption, perceived usefulness, security concerns, technical infrastructure, intent to use

## Abstract

**Background:**

This study evaluates the impact of e-health solutions on healthcare systems, focusing on how the perceived usefulness of these technologies affects healthcare workers’ intentions to use them.

**Methods:**

The study used a cross-sectional approach in the form of a close-ended questionnaire to collect quantitative data from a sample of 130 healthcare professionals randomly selected. The collected data was then analyzed using SPSS version 30, statistical analysis was utilized such as frequency test, reliability analysis, and correlation coefficient analysis**.**

**Results:**

The findings suggest a statistically significant correlation between attitudes toward e-health and intention to use, with a moderate effect. The implementation of e-health technologies has a positive impact on healthcare management, though the magnitude of the effect varies depending on the technology and context and prior computer expertise significantly influences health workers’ attitudes toward adopting and using e-health**.**

**Discussion:**

E-health technologies can significantly improve operational efficiency, reduce costs, and enhance the quality of care in healthcare system. Successful implementation requires careful planning, investment in infrastructure, addressing security concerns, and training of healthcare professionals.

## 1 Introduction

While e-health leverages information and communication technologies to enhance healthcare, its journey from potential to widespread, effective implementation remains complex (1–5). Despite WHO's emphasis and the need for national frameworks, many initiatives fall short of their projected impact (6–8).

Research extensively highlights e-health's capacity to improve efficiency, reduce costs, and enhance care (9–12). However, a critical review shows a nuanced reality. Beyond these benefits, less explored are potential adverse effects, like diminished physician attention due to digital interfaces (13). This reveals a critical gap: understanding the complexities of “proper implementation,” which dictate if e-health truly benefits or inadvertently harms patient-centered care and organizational efficiency. Furthermore, while economic analyses project e-health's benefits outweighing substantial costs, critical scrutiny shows long-term gains often hampered by unforeseen complexities, poor integration, and inadequate user adoption, questioning consistent return on investment realization (14).

Moreover, existing literature adequately catalogues e-health adoption barriers—including socioeconomic disparities, lack of user-oriented content, and critical interoperability issues (15–17). Yet, a deeper critical analysis reveals a persistent gap in understanding their interplay and the efficacy of mitigation strategies. Despite recognized potential for improved patient health literacy, cost-effective care, and enhanced disease management, and positive impact on total quality management, the field still grapples with fundamental implementation hurdles such as privacy concerns and complex physician-patient relationships in digital environments (18–20).

Crucially, a significant analytical imbalance pervades current literature. It disproportionately emphasizes digital health's technological aspects, critically neglecting its implications for strategic and operational management (21–24). This focus on technical capabilities, at the expense of real-world organizational impact, leaves a substantial void in our quantitative understanding of e-health's actual effects on healthcare systems. Additionally, a fundamental methodological limitation in much empirical work is its narrow scope, predominantly focusing on medical doctors (23, 25). This restricted perspective risks significant bias, providing an incomplete understanding of e-health integration within the multidisciplinary healthcare environment. It fails to capture the diverse experiences and adoption dynamics of a broader professional spectrum. Recent studies underscore the critical importance of understanding the perspectives of a wider range of healthcare professionals to truly “unlock the black box” of e-health adoption (26, 27).

Against this backdrop of analytical deficiencies and methodological constraints that the present study intervenes. By offering a robust quantitative analysis that specifically encompasses a broader spectrum of healthcare professionals, this research aims not merely to describe but to critically elucidate the actual impact of e-health on healthcare systems. This provides a more balanced perspective and substantially enhances the generalizability of findings crucial for effective implementation strategies. This study focuses on the following research questions:

1. How does perceived usefulness of e-health technologies impact healthcare workers**’** intention to use them?
2. To what extent do security concerns influence the adoption of e-health systems in healthcare organizations?
3. How does the availability of technical infrastructure affect healthcare workers**’** attitudes towards e-health?

## 2 Materials and methods

### 2.1 Study design, setting and participants

This cross-sectional study investigates and analyzes the factors influencing Saudi healthcare professionals' decision to adopt and use e-health technologies. A convenience sampling approach was employed and distributed through different social media platforms for healthcare professionals working in Saudi hospitals from various roles, including executives, doctors, nurses, and others. Participants were contacted online and informed that their participation was voluntary, and their responses would be treated confidentially, thereby obtaining informed consent. Data was collected through a structured questionnaire, and it utilized a five-point Likert scale, ranging from “strongly disagree” to “strongly agree,” to capture participants' perceptions of e-health technologies, their perceived usefulness, ease of use, and intention.

### 2.2 Data collection tools

The research utilized a quantitative questionnaire that contained close-ended questions in an electronic format and distributed using social media with a covering statement that highlights the objectives and importance of the survey and asked for the participants consent. The questionnaire items were adopted from multiple previous studies, and the reliability already tested where Cronbach alpha was reported at (0.899) (5, 28, 29). The survey included demographic questions and queries exploring participants' knowledge, perceptions, and experiences with e-health technology applications.

Respondents rated the survey questions on a 5-point Likert scale, ranging from “strongly disagree” to “strongly agree”. Additionally, participants' level of Information Technology literacy and experience was measured on a scale of “None = no IT literacy”, “Minimum = little IT literacy”, “Fairly = average IT literacy”, and “Maximum = sufficient IT literacy”. In total, 138 questionnaires were completed and retrieved, but 8 were excluded due to lack of consent, leaving 130 surveys for further analysis using SPSS software version 30.

### 2.3 Data analysis and management

The 130 completed questionnaire were then organized, and the data was entered into SPSS software for further analysis. Each question was examined using a coding system that categorized the responses into relevant themes. To analyze the data, the study employed a range of statistical techniques, including descriptive statistics, correlation and regression analysis.

## 3 Results

### 3.1 Demographic data

Table 1 displays the demographics of the 130 respondents, where the majority of respondents were Females at 66.9%. Also, profession wise doctors were the most respondents (56%) followed by 30% from various allied health professional backgrounds categorized as other, which demonstrates that the study covered various healthcare professionals' perceptions.

**Table 1:** Demographic data (*N* = 130).

Demographic data		Frequency	Percentage
Gender	Female	87	67%
	Male	43	33%
Profession	Physician	73	56%
	Nurse	18	14%
	Other	39	30%
Years of experience	1–5 Years	14	11%
	6–10 Years	22	17%
	11+ Years	94	72%
IT Knowledge	None	5	4%
	Minimum	29	22%
	Fair	54	42%
	Good	42	32%

The respondents had significant experience in the healthcare industry, with 72.3% having worked for over 11 years and a great number of the respondents consider themselves to have a fair understanding of IT, accounting for 41.5% of the sample, which implies that majority of healthcare professionals have moderate level of comfort with IT, which can influence their acceptance of e-health technologies.

### 3.2 Correlation

The results of Pearson Correlation analysis as displayed in Table 2 reveal insights into the relationships between key variables pertinent to e-health technology adoption and its impact on healthcare management.

**Table 2:** Correlation analysis.

Pearson correlation		Pu	Att	Intent	IT exp	Info shar	Sec	Techinfra
Pu	Pearson correlation	1						
	Sig. (2-tailed)							
Att	Pearson correlation	.602*	1					
	Sig. (2-tailed)	<.001						
Intent	Pearson correlation	.380*	.486*	1				
	Sig. (2-tailed)	<.001	<.001					
IT exp	Pearson correlation	.439*	.520*	.247*	1			
	Sig. (2-tailed)	<.001	<.001	.005				
Info shar	Pearson correlation	.371*	.464*	.275*	.457*	1		
	Sig. (2-tailed)	<.001	<.001	.002	<.001			
Sec	Pearson correlation	.377*	.545*	.244*	.498*	.542*	1	
	Sig. (2-tailed)	<.001	<.001	.005	<.001	<.001		
Techinfra	Pearson correlation	.229*	.389*	.269*	.491*	.392*	.584*	1
	Sig. (2-tailed)	.009	<.001	.002	<.001	<.001	<.001	

Pu, perceived usefulness; Att, attitude towards e-health; Inten, intention to use e-health; IT exp, staff IT experience; Info shar, information sharing; Sec, security concerns; Techinfra, technical infrastructure.

* Correlation is significant at the 0.01 level (2-tailed).

The results of the correlation among the 7 sections were all positively and significantly correlated. The section with the highest correlated value was the correlation between the variable perceived usefulness and the variable attitude towards e-health at (*r* = 0.602, *p* < 001), while the lowest correlated variable was the perceived usefulness and technology infrastructure (*r* = 0.229, *p* < 0.01).

### 3.3 Regression

Regression analysis as displayed in Table 3 was used to answer the research questions, to predict how perceived usefulness of e-health technologies impacts intention to use e-health, how security concerns influence the adoption of e-health systems in healthcare organizations and the impact of the availability of technical infrastructure on workers' attitudes towards e-health.

**Table 3:** Linear regression results for security concerns and its impact on the adoption of e-health systems.

Coefficients^a^	*R* square	Adjusted *R* square	Standardized coefficients beta	Sig.	Std. error of the estimate
Pu	.142	.136	.377	<0.001*	.926
Att	.297	.291	.545	<0.001*	.839
Intent	.060	.052	.244	<0.005	.970
IT exp	.248	.242	.498	<0.001*	.867
Info shar	.275	.269	.524	<0.001*	.852
Techinfra	.341	.336	.584	<0.001*	.812

^a^ Pu, perceived usefulness, Att, attitude towards e-health; Inten, intention to use e-health; IT exp, staff IT experience; Info shar, information sharing; Sec, security concerns; Techinfra, technical infrastructure.

* Correlation is significant at the 0.01 level (2-tailed).

#### 3.3.1 Perceived usefulness impact on intention to use e-health

Linear regression analysis indicated that perceived usefulness of e-health technologies was a significant predictor of intention to use e-health with a positive relationship. The standardized coefficient for perceived usefulness was 0.380, suggesting that a one standard deviation increase in perceived usefulness would result in a 0.380 standard deviation increase in intention to use e-health and the corresponding *p*-value of less than 0.001, indicating that the relationship between perceived usefulness and intention to use e-health was statistically significant.

#### 3.3.2 Security concerns impact on the adoption of e-health systems

Linear regression analysis indicated that security concerns was a significant predictor on the adoption of e-health systems with a positive relationship between all different aspects of the questionnaire with a significance of (*p* < 0.001) except of intention to use e-health where the significance was at (*p* < 0.005) as demonstrated in Table 3.

The factors perceived usefulness, attitude, intention, IT experience, information sharing, and technical infrastructure all significantly affect e-health adoption. Attitude and Technical infrastructure are particularly strong predictors.

#### 3.3.3 Impact of technical infrastructure on workers' attitudes towards e-health

Linear regression analysis indicated that technical infrastructure availability was a significant predictor of workers attitudes towards use of e-health with a positive relationship. The standardized coefficient for technical infrastructure was 0.390, suggesting that a one standard deviation increase in technical infrastructure would result in a 0.390 standard deviation increase in workers attitudes towards use of e-health and the corresponding *p*-value of less than 0.001, indicating that the relationship between technical infrastructure availability and workers attitudes towards use of e-health was statistically significant.

The result from the quantitative analysis reveals that the implementation of e-health technologies has a positive impact on healthcare systems.

## 4 Discussion

This quantitative analysis provided critical insights into the multifaceted dynamics of e-health adoption and its tangible effects on healthcare systems. This investigation highlights the complex interplay among technological preparedness, organizational structures, and individual cognitive and emotional reactions, all of which collectively influence the course of e-health implementation (11, 30). Despite the acquisition of e-health systems, implementation is often limited to pilot projects, lacking the comprehensive coordination and scalability necessary for widespread efficacy (5, 31).

### 4.1 Perceived usefulness and behavioral intention

The current research findings strongly affirm that the perceived usefulness of e-health technologies is a significant predictor of healthcare workers' intention to use them, demonstrating a positive relationship. This aligns consistently with established technology acceptance models that posit perceived usefulness as a primary determinant of behavioral intention in technology adoption (5, 28).

Indeed, a review of the literature indicates that perceived usefulness significantly influences the intention to adopt and use healthcare technology, with users more likely to embrace and integrate technology into their daily practice when they perceive it as beneficial (27, 31). Healthcare professionals are inherently pragmatic; they are more inclined to adopt tools that they perceive as directly enhancing their efficiency, improving patient care quality, or streamlining their workflows (32).

For example, perceived usefulness in healthcare often translates to improved patient care, faster service delivery, better documentation, and accurate, low-cost medical monitoring (26). The strong correlation observed suggests that when e-health applications offer clear, tangible benefits—such as improved accuracy, reduced documentation time, or enhanced information transfer—users are more likely to integrate these technologies into their daily practice. This reinforces the critical importance of demonstrating the clear advantages and value proposition of e-health solutions to foster acceptance among end-users.

The findings suggest that a successful e-health strategy necessitates a multi-pronged approach that addresses technological, organizational, and human factors. The emphasis on training programs is particularly relevant, given the crucial role of continuous education in facilitating ease of use and ensuring that healthcare professionals can effectively leverage new IT systems (16). Moreover, the importance of robust technical infrastructure cannot be overstated, as its availability directly impacts workers' attitudes towards e-health (33).

### 4.2 Security concerns and e-health adoption

The current research in its regression revealed that security concerns significantly influence the adoption of e-health systems. This finding resonates with broader literature, which consistently identifies privacy and confidentiality as paramount barriers in healthcare technology implementation (16, 34). Privacy concerns have a significant impact on technology acceptance, as individuals are increasingly worried about the security of their personal information (35). The highly sensitive nature of patient data necessitates robust security protocols and transparent communication regarding data privacy. Doubts about data privacy and security, coupled with insufficient digital skills, can hinder technology acceptance (35).

Healthcare professionals, being entrusted with confidential patient information, are naturally hesitant to adopt systems perceived as vulnerable to breaches or misuse. While security concerns are present, our results indicate that addressing them effectively through stringent data protection measures and transparent policies can foster greater trust and facilitate adoption. Moreover, the results emphasize the importance of aligning e-health interventions with broader health strategies, indicating that successful e-health initiatives are those that are integrated into the existing healthcare ecosystem. This integration requires a collaborative approach involving various stakeholders, including healthcare professionals, IT specialists, policymakers, and patients, to ensure that e-health solutions are contextually relevant and aligned with the needs of the target population (35, 36).

Conversely, a failure to alleviate these concerns can lead to significant resistance, regardless of other perceived benefits (5). Ensuring privacy of patient information and implementing strong government regulations for health data protection are critical factors that can increase the adoption of e-health systems (37). Such concerns also impede medical record portability and can reduce the perceived usefulness and ease of use of e-health tools (26).

### 4.3 Technical infrastructure and attitudes towards e-health

The availability of a robust technical infrastructure was found to be a significant predictor of workers' attitudes towards e-health, demonstrating a positive relationship. This underscores a fundamental prerequisite for successful e-health implementation: a reliable and accessible technological backbone is not merely a logistical requirement but a critical determinant of user acceptance and positive attitudes. Technical infrastructure, defined as the foundational IT components of an organization's IT service, directly influences attitudes and intentions towards e-health (32).

Similar studies confirm that poor infrastructure, including issues with hardware, software, and networking, significantly impedes e-health adoption and utilization (5). An unreliable or inadequate infrastructure inevitably leads to user frustration, operational inefficiencies, and a diminished perception of e-health's overall utility. Inadequate training and infrastructure are major barriers to digital readiness and capability (35).

Therefore, sustained investment in, and maintenance of, high-quality technical infrastructure is essential to cultivate a conducive environment for e-health integration and positive user experience, and improving hospital's technical infrastructure should be a priority for implementers (16, 32). Lack of health information exchange and data interoperability are frequently cited obstacles, suggesting that better integration and availability of patient information across systems could further promote e-health assimilation (26).

Furthermore, perceived usefulness, alongside technical infrastructure, often exhibits a stronger effect on user attitude and behavioral intention compared to perceived ease of use, underscoring its crucial role in e-health system adoption (32).

### 4.4 Organizational readiness

Despite the acquisition of e-health systems, the current research, consistent with other observations, notes that implementation is often limited to pilot projects, lacking the comprehensive coordination and scalability necessary for widespread efficacy. This highlights a crucial aspect of organizational readiness—the capacity of an organization to successfully implement and sustain change. The organizational readiness is crucial for successful implementation, with factors such as top management support, supervisor assistance, and peer influence playing significant roles (11). Healthcare professionals' readiness and competency are critical for implementation success, but budget constraints, lack of IT staff, and perceptions of systems as time-consuming remain major barriers (12).

The gap between anticipated and demonstrated benefits of e-health applications often stems from an insufficient consideration of the complex interplay between social and technical elements during implementation. Factors such as a lack of political support, insufficient funding, and weak cooperation among stakeholders can impede an organization's readiness for change (9). Furthermore, neglecting the human element, including healthcare workers' expectations and willingness to adapt, can be counterproductive (9), leading to resistance and jeopardizing initiatives (16). This resistance often stems from a reluctance to change from accustomed paperwork systems to new technological approaches, and concerns regarding failure (16).

Organizational complexities, including workload distribution, reward mechanisms, and staff training, are frequently cited barriers (3, 16). Strong leadership and effective change management are crucial for overcoming implementation barriers, emphasizing that e-health adoption is a socio-technical transformation (12, 27). Past experience with e-health platforms, whether successful or failed, can also alter perceived benefits and act as a demotivating factor for future adoption (3).

Successful implementation therefore requires not just technological preparedness but also robust change management strategies that address human factors, fostering an environment where organizational members are willing and able to adjust and maintain continuity with new systems (9).

In conclusion, the current research reinforces that a successful e-health strategy necessitates a multi-pronged approach that addresses technological, organizational, and human factors. The regression analysis further elucidates the predictive power of perceived usefulness, security concerns, and technical infrastructure on e-health adoption, offering actionable insights for policymakers and healthcare administrators (11, 38). The emphasis on continuous training programs, robust technical infrastructure, and stringent data protection measures is pivotal for fostering adoption. Moreover, aligning e-health interventions with broader health strategies and tailoring them to specific contextual needs, rather than adopting a “one-size-fits-all” approach, is essential for realizing the transformative potential of e-health in improving access, quality, and efficiency within healthcare systems.

## 5 Conclusion

The study's main findings indicate a statistically significant positive correlation between healthcare workers' attitudes toward e-health and their intention to use it, with prior IT expertise also influencing these attitudes. Perceived usefulness, security concerns, and technical infrastructure were identified as significant predictors of e-health adoption, with perceived usefulness showing a positive relationship with intention to use, and technical infrastructure positively impacting attitudes. Overall, e-health technologies were found to positively impact healthcare management, improving operational efficiency, reducing costs, and enhancing quality of care.

These implications for healthcare practice suggest that successful e-health implementation requires strategic planning, robust infrastructure investment, and demonstrating clear benefits to professionals. Furthermore, continuous training, stringent data security measures, and aligning e-health interventions with broader health strategies are crucial, with an emphasis on context-specific approaches rather than a “one-size-fits-all” method.

However, the study faced limitations due to its convenience sampling method, which restricts the generalizability of findings to the broader Saudi healthcare worker population, and its cross-sectional design, preventing causal inferences.

Consequently, recommendations for future research include utilizing longitudinal designs to investigate long-term effects and causal relationships, focusing on context-specific e-health initiatives, deepening research into security and ethical parameters, and exploring agile strategies to adapt to evolving healthcare landscapes.

## Data Availability

The raw data supporting the conclusions of this article will be made available by the authors, without undue reservation.
